# Finding a Needle in a Haystack: An Unusual Case of Foreign Body Aspiration

**DOI:** 10.7759/cureus.78109

**Published:** 2025-01-27

**Authors:** Annette Mathew, Taraneh Honarparvar, Austin Trent, Alejandro Biglione

**Affiliations:** 1 College of Medicine, Dr. Kiran C. Patel College of Osteopathic Medicine, Nova Southeastern University, Davie, USA; 2 Internal Medicine, Wellington Regional Medical Center, Wellington, USA

**Keywords:** airway foreign body, body mri, chest radiography, foreign body aspiration, metallic foreign body, pulmonary atelectasis, sewing needle

## Abstract

Foreign body aspiration can have serious medical consequences ranging from asphyxiation to infection. Aspiration of sharp, metallic foreign bodies can also pose risks of vascular injury and damage to the surrounding tissue. On the other hand, in the presence of a foreign body, it should be noted that imaging techniques such as magnetic resonance imaging (MRI) may be contraindicated as they can lead to serious injury when the foreign bodies are brought under a magnetic field. We present a case of an incidental foreign body identified on chest radiography that prevented a brain MRI in a patient who had a history of sewing needle aspiration as a child, with its presence in the left thoracic cavity since that time.

## Introduction

Foreign body aspiration can have serious consequences such as asphyxiation, respiratory failure, atelectasis, post-obstructive pneumonia, lung abscess, and sepsis [1]. Approximately 3,000 deaths occur every year due to foreign body aspiration. Many of these deaths occur before patients arrive at the hospital for treatment and care [2].

It is believed that foreign body aspiration typically presents in the right lower lobe of the lung due to its more vertical presentation and larger diameter of the bronchus. Because of the consequences of this very serious event, management may include bronchoscopy when a foreign body is identified upon radiologic imaging. However, there is no truly defined standard of care for this event [1].

Eighty percent of foreign body aspiration events in the pediatric population occur in patients under the age of three [3]. Meanwhile, the incidence of foreign body aspiration in adults is 0.66 per 100,000 individuals [4]. Some patients may remain asymptomatic from a foreign body aspiration event and a metallic foreign body can be found incidentally when a radiologic test is performed for another indication. However, all radiologic imaging studies should be carefully chosen, as they have the potential to impact patient safety and care.

Patients are routinely screened for implantable devices, hearing aids, piercings, and more because these devices are attracted to the magnetic force exerted by MRI machines. This attraction can result in a projectile effect, displacement, or malfunction [5]. Having a metallic foreign body retained in the lung can have serious consequences when a patient undergoes an MRI. Aspirated metallic foreign bodies may prevent the use of MRI as a diagnostic tool because they may be displaced and cause tissue damage when exposed to a high magnetic field [5].

## Case presentation

In this case report, we present a 54-year-old male with a medical history of hyperlipidemia, coronary artery disease, and coronary stent placement, who presented to the Emergency Department (ED) after experiencing a syncopal episode and altered mental status. His wife found him unconscious, and no seizure activity was witnessed. A comprehensive history could not be obtained due to the patient’s altered mental status.

The patient’s vital signs were within normal limits: temperature 97.3°F, pulse 77 bpm, blood pressure 125/79 mmHg, respiratory rate 17 breaths per minute, and oxygen saturation 96% on room air. On physical examination, the patient was confused, had slurred speech, and was oriented to person. He had a full range of motion in all extremities with no focal deficits.

Initial laboratory studies included a complete blood count (CBC), which revealed normocytic normochromic anemia with a hemoglobin level of 12.3 mg/dL and a hematocrit of 36.8%. The basic metabolic panel was normal except for an elevated blood glucose level of 248 mg/dL. Additionally, his urine drug screen was negative.

**Figure 1:**
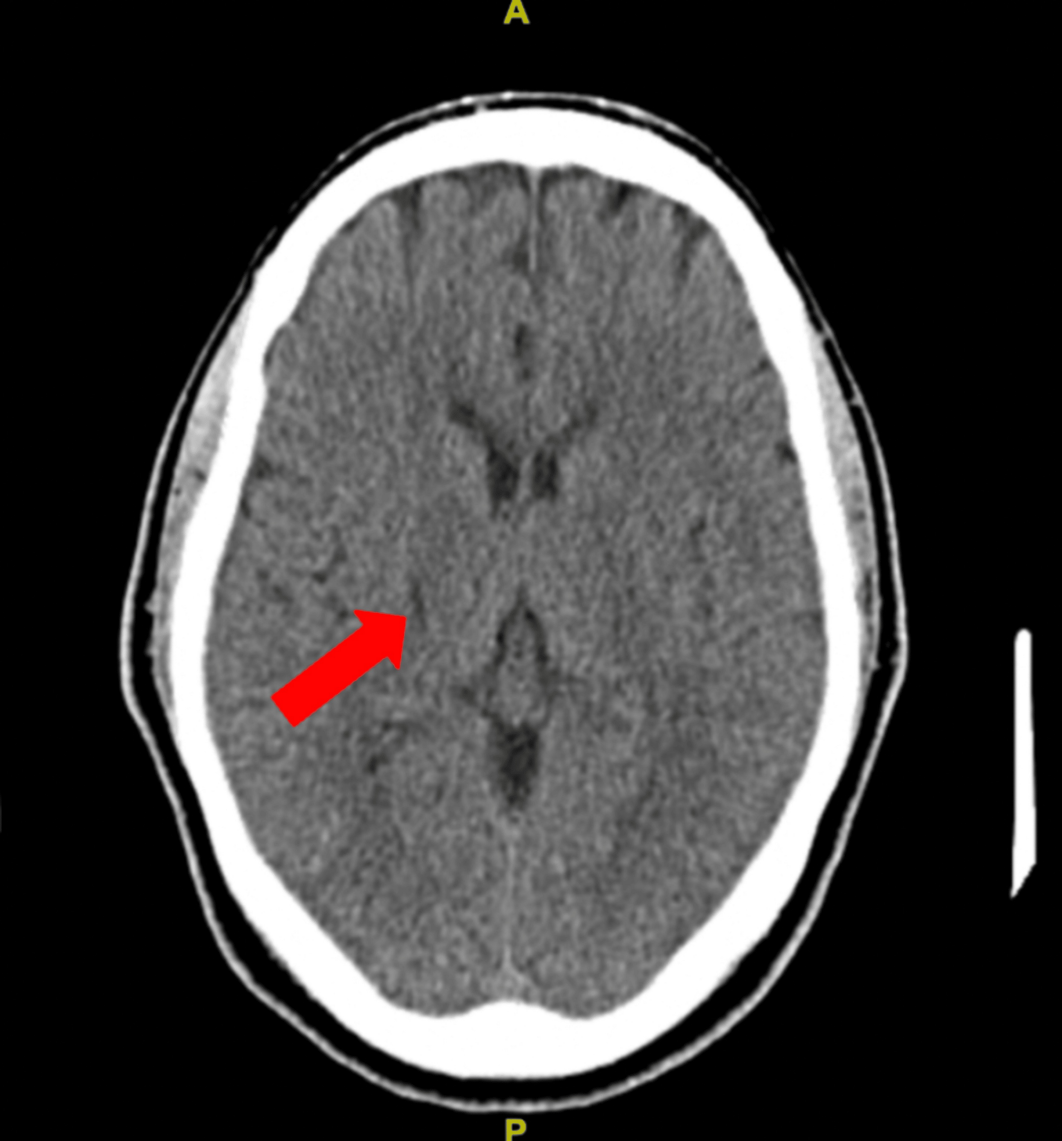
Small ovoid/linear hypodensity in the right gangliocapsular region representative of chronic infarct.

A computed tomography (CT) scan of the head revealed a small ovoid/linear hypodensity in the right gangliocapsular region, consistent with a chronic infarct (Figure 1).

**Figure 2:**
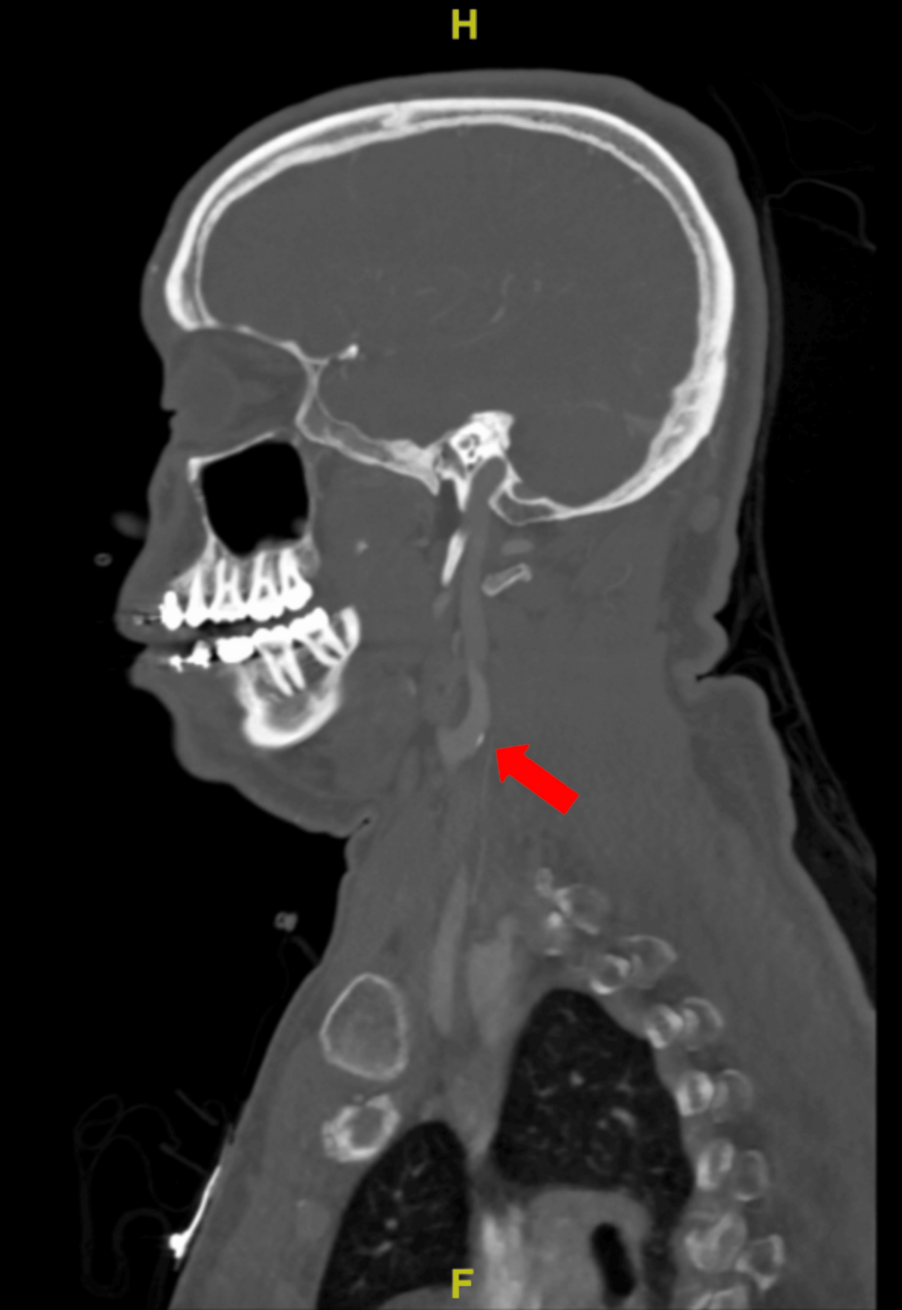
Small calcified atheromatous plaques on the left carotid bulb.

**Figure 3:**
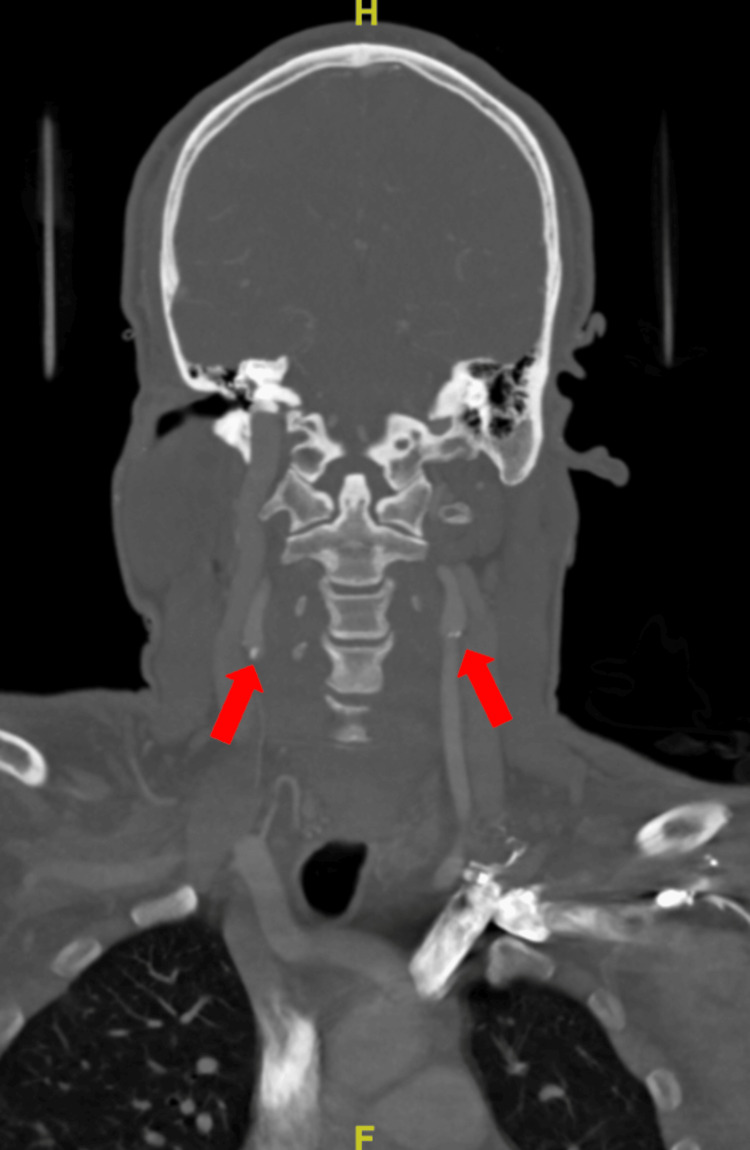
Small calcified atheromatous plaques on origins of bilateral cervical ICAs without luminal narrowing. ICAs, internal carotid arteries

A CT angiogram of the head revealed small calcified atheromatous plaques on the left carotid bulb and origins of bilateral cervical internal carotid arteries (ICAs) without luminal narrowing. Additionally, calcified atheromatous plaques were observed in the cavernous segments of bilateral ICAs with mild luminal narrowing (Figure 2 and Figure 3).

**Figure 4:**
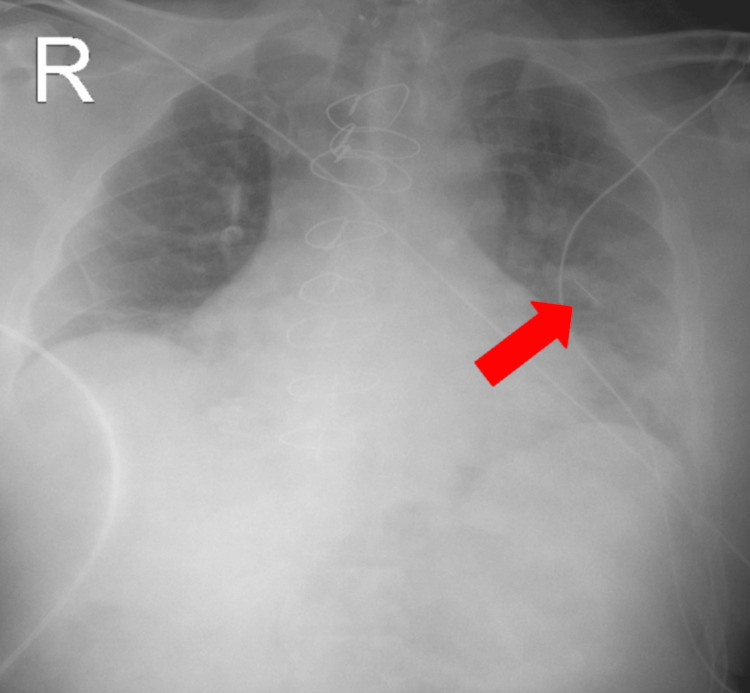
Linear foreign body between intercostal spaces four and five.

**Figure 5:**
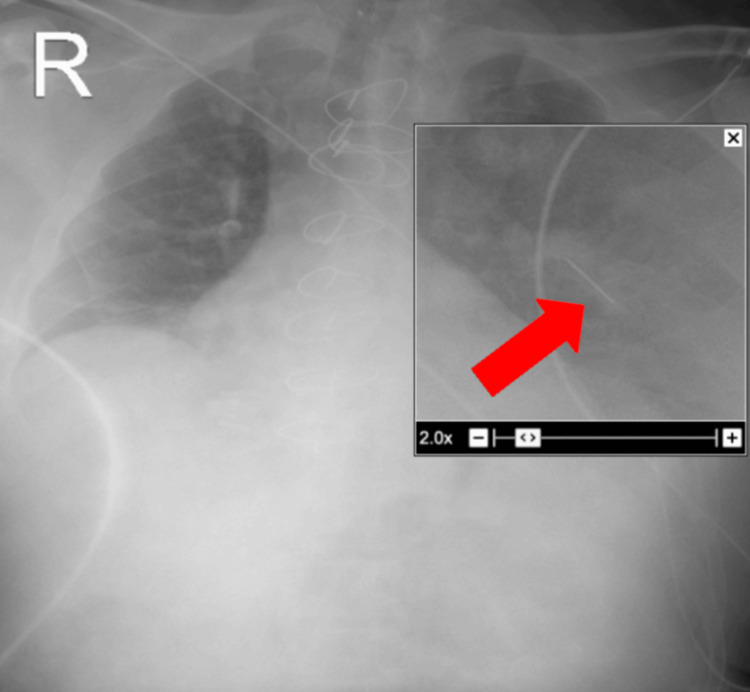
Enhanced image of the linear foreign body between intercostal spaces four and five.

An electrocardiogram (EKG) showed normal sinus rhythm. Finally, a chest radiograph revealed a linear foreign body between intercostal spaces four and five, which was an incidental finding (Figure 4 and Figure 5).

An MRI of the brain would have been ordered to further evaluate for stroke; however, the foreign body noted on the chest radiograph precluded the safe use of MR imaging in this patient, and its use was postponed pending further information.

Upon improvement of his mentation, further history could be obtained. The patient became more alert and oriented. When asked about the needle in his chest radiograph, he recalled being told he had "swallowed a sewing needle as a child." However, the patient had remained asymptomatic throughout his life and only recalled the event when informed about the needle in his chest radiograph. Upon discharge, the patient was recommended to follow up with a pulmonologist; however, he was lost to follow-up.

## Discussion

Aspiration of a foreign body can have serious consequences such as asphyxiation, respiratory failure, atelectasis, post-obstructive pneumonia, lung abscess, and sepsis. Some patients do not experience any symptoms and suffer no deleterious complications. In fact, they may remain asymptomatic for life, with the foreign object only discovered incidentally during radiologic studies performed for other indications. Metallic foreign objects may preclude the use of MRI as a diagnostic tool because they can cause tissue damage by being displaced when exposed to a high magnetic field.

In our case, a needle was incidentally found in the lung parenchyma during chest radiography. The clinical implication was that performing a brain MRI could be contraindicated due to the risk of lung injury. This risk arises from the magnetic field strength of the MRI machine, which could displace the metallic foreign body into sensitive tissue. Pneumothorax, hemothorax, empyema, lung abscess, and broncho-esophageal fistula have all been listed as major complications [1]. We reviewed the existing literature to determine whether an MRI would be contraindicated in the presence of a needle within the chest cavity. The literature suggests the immediate removal of the needle from the parenchyma, as it may damage the vasculature. Rigid bronchoscopy has been recommended as both a diagnostic and therapeutic modality due to its ability to ventilate the lung and provide better visibility of foreign objects. However, if rigid bronchoscopy is not feasible, flexible bronchoscopy is the preferred procedure [1].

There are also concerns regarding needles within the venous system, which pose a high risk of migration to the cardiopulmonary system during MRI. The needle could be pulled by the MRI's strong magnetic field and migrate to the right side of the heart or the lungs [6]. As a result, an MRI study is contraindicated in patients with metallic objects embedded in sensitive tissues, as this could cause damage to surrounding structures. Absolute contraindications for MRI include certain cardiac implantable devices, metallic intraocular foreign bodies, implantable neurostimulation systems, cochlear implants, catheters with metallic components, metallic dental implants, and piercings [5].

In some cases, MRIs have been performed without knowledge of embedded foreign bodies, and no significant issues occurred due to reactive fibrosis anchoring the objects to the tissue and preventing migration [6]. While the strength of the magnetic field (in this case, 1 Tesla) may theoretically influence complications, no studies have precisely elucidated this issue.

When imaging studies are limited by metallic foreign bodies, there are other imaging modalities that may be useful in emergent situations. CT scans may prove useful for localizing metallic foreign bodies and complications such as abscesses or vascular injury associated with these bodies [7]. Another imaging modality that may be used is ultrasound. This requires no radiation exposure and can be used in a wider variety of patients, such as pediatric and pregnant patients. Ultrasound has proven to be useful in showing radiolucent objects such as plastic. However, this imaging modality may be limited due to operator-dependent results; additionally, ultrasound may not be able to penetrate deeper structures or images through bones or in this case, air-filled cavities like the lungs [8]. Finally, an X-ray is the first line modality to image foreign bodies. It is advantageous for its effectiveness in detecting metallic foreign bodies and radiodense objects. However, it may be limited due to the two-dimensional nature of imaging, which does not allow for accurate localization of foreign bodies [9]. Regardless, it remains the superior imaging modality in cases of metallic foreign bodies.

Upon reviewing the literature, we found a case involving a 55-year-old patient who aspirated a sewing needle 15 years prior, later discovered on a chest radiograph. The needle was found perpendicular to the left cardiac border, with its tip embedded in the border. The patient refused additional imaging or resection and later died from vascular compromise associated with the needle [10].

Finally, our case was unusual, as foreign bodies are typically found in the right main bronchus due to their anatomy. However, in this patient, the needle was found in the left lung parenchyma [1].

## Conclusions

The incidental finding of foreign bodies on imaging can serve as a teaching point for patient safety. It is important for patients to understand the risks associated with the presence of metallic foreign bodies within the body and the contraindications of certain imaging modalities, which may cause harm by displacing the foreign body. This case also raises the question of why this patient did not experience any symptoms associated with the aspiration of a sewing needle and how he managed to remain asymptomatic throughout his life.

The aspiration of foreign bodies is not a rare occurrence and can happen even in patients without significant risk factors, such as dysphagia. Aspiration of sewing needles can have serious consequences. Additionally, incidental findings of needles within the thoracic cavity can have clinical implications during MRI. Patients should be informed of these risks and understand the importance of foreign body removal before undergoing this type of imaging.
